# Supplementary material for: Nutrients Supplementation through Organic Manures Influence the Growth of Weeds and Maize Productivity
Source: Molecules. 2020 Oct 24;25(21):4924. doi: 10.3390/molecules25214924 (PMC7663199; doi:10.3390/molecules25214924)
Supplement: Supplementary file 1 [file molecules-25-04924-s001.pdf]

Supplementary Materials

Nutrients Supplementation through Organic Manures Influence the Growth of Weeds and Maize Productivity

Supplementary Table S1. Nutrient content (%) in different organic manures.

Year of experiment	Organic manures	Nitrogen (N)	Phosphorus (P_2O_5)	Potassium (K_2O)
Year 1	Vermicompost	1.56	0.51	1.07
	Farmyard manure	0.65	0.26	0.41
	Brassicaceous seed meal	4.82	1.76	1.22
	Neem cake	5.23	1.09	1.44
Year 2	Vermicompost	1.54	0.52	0.99
	Farmyard manure	0.59	0.25	0.43
	Brassicaceous seed meal	4.80	1.77	1.21
	Neem cake	5.18	1.07	1.43

Supplementary Table S2. Physico-chemical properties and nutrient status of post-harvest soil (after first year of maize crop).

Treatments combination		pH	EC (dS/m)	Organic carbon (g/kg)	Nutrient status (kg/ha)		
					N	P_2O_5	K_2O
Weedy check	RDF	7.15	0.223	4.25	189	36.7	145
	RDF + FYM	7.17	0.247	5.52	206	35.8	141
	RDF + VC	7.22	0.210	4.20	188	36.2	159
	RDF + BSM	7.44	0.150	3.48	169	40.1	153
	RDF + NC	6.98	0.297	4.16	186	41.8	137
Chemical weed management	RDF	7.64	0.273	3.64	191	41.8	142
	RDF + FYM	7.02	0.227	4.88	195	39.8	158
	RDF + VC	7.04	0.347	4.25	189	41.8	157
	RDF + BSM	6.99	0.247	4.40	193	36.7	152
	RDF + NC	7.40	0.267	4.59	198	38.2	149
Integrated weed management	RDF	7.26	0.287	4.25	189	42.1	154
	RDF + FYM	6.98	0.210	4.35	192	36.1	152
	RDF + VC	7.18	0.223	4.35	192	42.4	151
	RDF + BSM	7.03	0.297	4.83	204	34.3	145
	RDF + NC	7.20	0.213	3.96	181	45.8	152
SEm ±		0.16	0.038	0.39	9	2.9	5
CD ($p \leq 0.05$)		NS	NS	NS	NS	NS	NS
